# Behavioural expression of ictal embarrassment: a stereo-EEG case report

**DOI:** 10.3389/fnhum.2026.1951958

**Published:** 2026-10-06

**Authors:** A. Scarabello, V. Pelliccia, F. Bisulli, M. Revay, F. Gozzo, A. Mingarelli, C. Peretto, R. Mai, C. Pizzanelli, F. Pizzo, F. Bartolomei, L. Tassi

**Affiliations:** 1 "Claudio Munari" Epilepsy Surgery Center, ASST Niguarda Hospital, Milan, Italy; 2 DIBINEM, Department of Biomedical and Neuromotor Sciences, University of Bologna, Bologna, Italy; 3 Epilepsy Program, IRCCS Istituto Delle Scienze Neurologiche di Bologna, European Reference Network for Rare and Complex Epilepsies (EpiCARE), Bologna, Italy; 4 Neurology Unit, University Hospital of Pisa, Pisa, Italy; 5 Institut de Neurosciences des Systèmes, Aix-Marseille Université, Marseille, France

**Keywords:** case report, direct electrical stimulation, functional connectivity, ictal embarrassment, bodily context

## Abstract

Embarrassment is a rare ictal manifestation whose neurophysiological basis remains poorly understood. We describe the case of a 40-year-old woman with drug-resistant focal epilepsy characterized by an unusual ictal semiology combining an intense positive emotional experience with behavioral manifestations of embarrassment. Stereo-EEG demonstrated seizure onset within the middle frontal gyrus bank of the superior frontal sulcus (F2sb), with rapid propagation to the dorsal anterior cingulate cortex (dACC) and superior anterior insula (sAI). Electrical stimulation of the F2sb reliably reproduced the habitual electroclinical seizure. While identical stimulation parameters elicited the subjective and autonomic manifestations in both supine and upright postures, the complete behavioral repertoire of embarrassment emerged only when stimulation was delivered in the standing position. Exploratory functional connectivity analyses revealed that overt embarrassment was associated with reduced node strength within a network encompassing the dACC, sAI, mesial prefrontal cortex (mPFC), and dorsolateral prefrontal cortex (dlPFC). More restricted network changes were associated with only mild sensations, whereas asymptomatic stimulation produced a distinct and partly reversed connectivity pattern. This case suggests that the behavioral expression of ictal embarrassment arises from the transient reconfiguration of a frontal-cingulo-insular network and is critically shaped by bodily context.

## 1 Introduction

Since Darwin’s seminal observations in *The Expression of the Emotions in Man and Animals*, emotions have been recognized as a crucial means of communication for managing interpersonal relationships (Crozier, 1998). Social interactions are essential for health and wellbeing in humans and other group-living primates (Perini et al., 2018). Compared with our closest hominoid relatives, humans have become increasingly reliant on cooperation for survival, fostering the evolution of socially meaningful emotions that facilitate group acceptance (Landers and Sznycer, 2022). Among these, embarrassment displays the hallmarks of an adaptive social emotion (Landers and Sznycer, 2022; Crozier and De Jong, 2012), remaining consistent across cultures and occurring even in congenitally blind individuals, despite variability in its elicitors (Landers and Sznycer, 2022).

In accordance with James’s somatic theory, embarrassment is accompanied by characteristic autonomic changes, including blushing (Pelliccia et al., 2022). Emotion and interoception share a common neural substrate in the anterior cingulate cortex (ACC; Pelliccia et al., 2022). Supporting this view, experimental studies show that lesions in area 24 of the ACC in infant monkeys impair separation crying when they are removed from their mothers (Devinsky et al., 1995).

Embarrassment may also emerge as a pathological phenomenon during epileptic seizures, likely reflecting disinhibition of encoded behavioural programs, which often manifest with motor and non-motor features in an exaggerated or caricatured manner (Tassinari et al., 2009).

Embarrassment has been reported as an ictal manifestation in only three cases. The first, described in 1982, involved a subject experiencing “embarrassment as though he had made a very foolish remark”, which resolved following resection of a mesial prefrontal astrocytoma (Devinsky et al., 1982). Another individual reported “shame and embarrassment as if he had done something wrong in public” during seizures, which ceased after removal of the right anterior frontal lobe (Lacazette et al., 2019). More recently, stereo-EEG localized the epileptogenic zone to the left ACC in a patient who described an aura “as if her thoughts were being intruded upon and her secret romantic interest exposed” accompanied by concordant behaviours such as blushing and avoidance of eye contact (Sun et al., 2021).

Although emotional experience, behavioural expression, and autonomic manifestations are generally assumed to form a coherent emotional response, they are subserved by partially distinct neural systems (Underwood et al., 2021) and may dissociate (Gross et al., 2000; Evers et al., 2014). During gelastic seizures, for instance, laughter might occur in the absence of mirth (Arroyo et al., 1993; Caruana et al., 2015). Additionally, social emotions, such as embarrassment, that integrate self-evaluation, social feedback, and moral cognition, may intrinsically involve internal conflict between subjective feeling and overt behaviour (Fourie et al., 2014).

We present the clinical history and stereo-EEG investigation of a patient with focal drug-resistant epilepsy who exhibited clear behavioural manifestations of embarrassment despite reporting a positively valenced emotional state. Intracerebral recordings and direct electrical stimulation provided a unique opportunity to explore the neural mechanisms underlying this rare semiology. As embarrassment is intrinsically shaped by social and bodily context, we examined whether body posture influenced the overt manifestations elicited by electrical stimulation. We further performed exploratory functional connectivity analyses to determine whether distinct clinical responses were associated with specific patterns of transient network reconfiguration (Bartolomei et al., 2019). By combining spontaneous seizures, electrical stimulation, posture-dependent behavioural modulation, and network analyses, we investigated how distributed brain networks generate the behavioural expression of ictal embarrassment.

## 2 Case description

A 40-year-old right-handed woman, with no family history of epilepsy and normal psychomotor development, had experienced seizures since the age of 11. She described an intense feeling of joy, which she likened to what she had felt at “the birth of her nephew”, followed by a sensation of heat rising from the chest to the face, accompanied by tachycardia. She explicitly denied any orgasmic or mystical quality to her emotional experience. During wakeful seizures, she displayed facial flushing and subtle postural adjustments. She was able to announce seizure onset verbally but responded only minimally to questions during episodes. When standing, she tended to distance herself physically from nearby people. Sleep-related seizures were characterized by complex integrated hyperkinetic movements, including trunk pronation and pelvic rocking. Despite multiple antiseizure medications, she continued to experience several seizures daily. The longest period of seizure freedom (5 years) was achieved early in the disease course with carbamazepine. Scalp EEG suggested seizure onset in the left mesial frontal region, whereas brain MRI and FDG-PET were inconclusive.

## 3 Diagnostic assessment

### 3.1 Stereo-EEG findings

At the age of 39, the patient underwent implantation of intracerebral electrodes targeting the left fronto-temporal regions, including the cingulate cortex, insula, dorsolateral, mesial, and orbitofrontal prefrontal cortices, temporal pole, and anterior hippocampus (Figure 1, panel A). Stereo-EEG recordings revealed subcontinuous spike-and-wave activity without a physiological background rhythm, suggestive of focal cortical dysplasia type II, in the middle frontal gyrus bank bordering the superior frontal sulcus (F2 sulcal bank, F2sb; Figure 1, panel B), and frequent low-amplitude spikes over the dorsal ACC (dACC; Figure 1, panel C).

**Figure 1:**
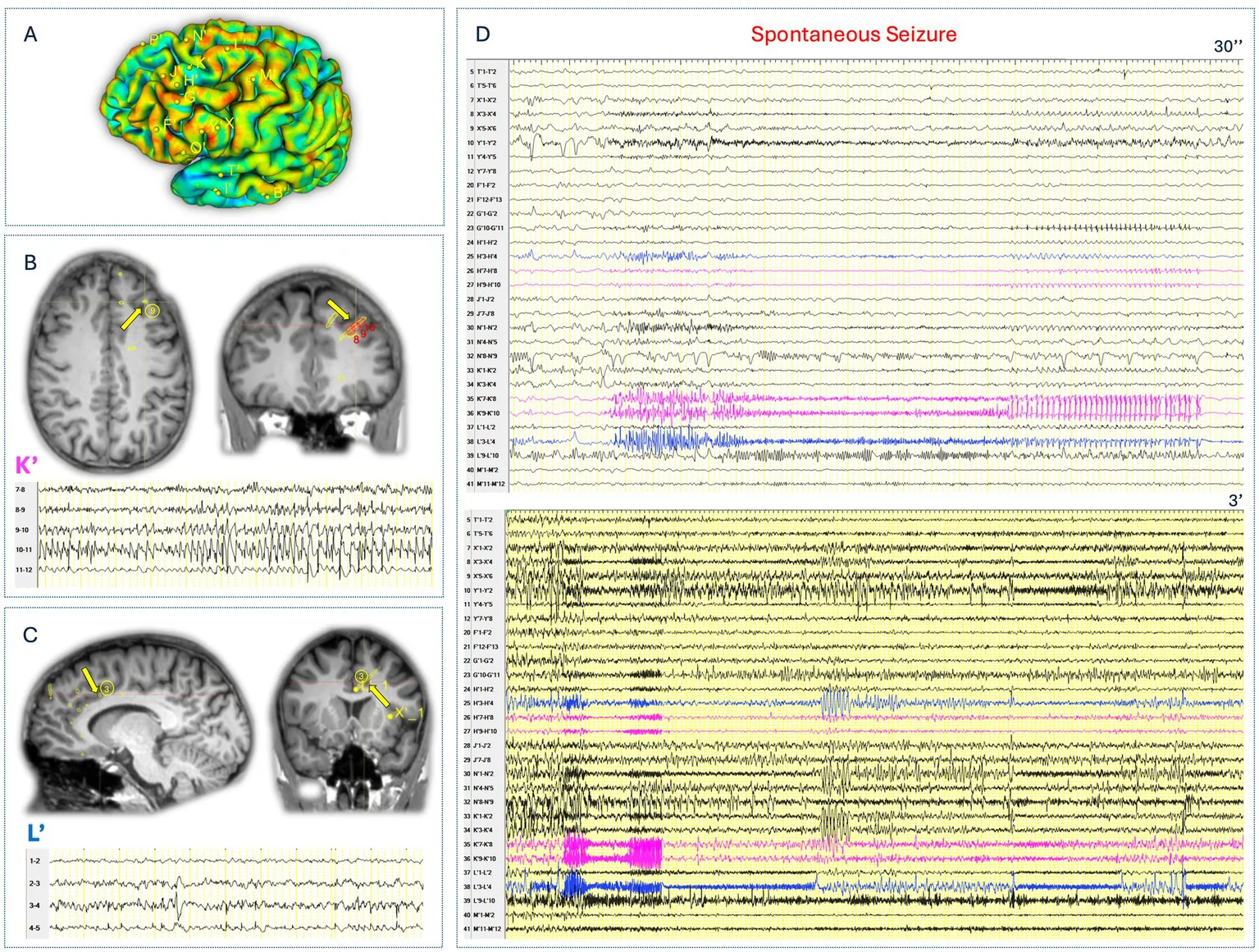
Anatomical localization and stereo-EEG features of the epileptogenic network. Panel **(A)**: stereo-EEG implantation scheme displayed on a 3D brain model with co-registered FDG-PET reconstruction. Panel **(B)**: anatomical localization of intermediate contacts of electrode K′ on T1-weighted MRI, coursing through the middle frontal gyrus bank bordering the superior frontal sulcus (F2sb). Corresponding stereo-EEG activity is shown below (filters: 30 𝜇V/mm, TC 0.1 s, HF 300 Hz, 30 s/page), with contacts 8–10 showing interictal patterns consistent with focal cortical dysplasia type II. Panel **(C)**: anatomical localization of the deep contacts of electrode L’ on T1-weighted MRI, sampling the dorsal and posterior portions of the ACC and the adjacent mesial prefrontal cortex, and exhibiting repetitive low amplitude spikes over a physiological background rhythm on stereo-EEG monitoring (filters: 30 𝜇V/mm, TC 0.1 s, HF 300 Hz, 30 s/page). Panel **(D)**: Stereo-EEG recording of a spontaneous seizure (top: 30 s/page, 50 𝜇V/mm, TC 0.03 s, HF 300 Hz; bottom: 3 min/page, 50 𝜇V/mm, TC 0.1 s, HF 300 Hz). Following an initial sequence of fast spiking activity involving the F2sb (intermediate contacts of electrodes K′ and H′, shown in pink), the dorsal ACC and adjacent mesial prefrontal cortex (deep contacts of electrode L’ and H′, shown in blue), and superior anterior insula–anterior short gyrus (sAI; deep contacts of electrodes X’ and Y′), low-voltage fast activity emerged and evolved into rhythmic spike–wave discharges. After termination of the discharge in the F2sb, low- to medium-voltage fast activity persisted independently within the dACC, mesial prefrontal cortex, and sAI, progressively shortening in duration.

The spontaneous seizure originated with low-voltage fast activity in the F2sb, with rapid propagation to the ipsilateral dACC and superior anterior insula (anterior short gyrus; sAI; Figure 1, panel D, top). Following termination of the discharge in the F2sb, tonic activity persisted transiently in the dACC and, to a lesser extent, in the sAI, coinciding with the re-emergence of mild, subjective manifestations and representing the terminal phase of the seizure (Figure 1, panel D, bottom).

### 3.2 Direct cortical stimulation

Electrical stimulation was systematically performed in a bipolar fashion to reproduce habitual seizures and map functional anatomy, using the following parameters: frequency 50 Hz, duration 5 s, pulse width 0.5 msec, and intensity between 1 and 4 mA. During electrical stimulation, the patient was fully awake and was instructed to keep her arms raised while reading words presented in front of her. Whenever clinically appropriate, particularly at sites evoking the habitual seizure, stimulation was performed under different conditions, in both the supine and upright positions, to evaluate whether bodily context influenced the expression of stimulation-induced motor signs. A summary of the stimulation trials across regions involved in the spontaneous seizure is reported in Table 1.

**Table 1:** Summary of stimulation trials across the frontal-cingulo-insular network.

Anatomical target		Contact pair	Intensity (mA)	Clinical response		Body posture	EEG response		FC
				Features	Category			AD details	
Prefrontal cortex	dlPFC (F2sb) [FCD IIa]	K’ 9–10	1	×	0		−		
			2	++	2		S		
			2	+++	3	↑	S		✓
			3	++	2		S		
			3	+++	3	↑	S		
		K’ 8–9	3	++	2		S		
			3	+++	3	↑	S		*
		K’7–8	1	×	0		−		
			2	×	0		−		
			3	×	0		AD	8 s [K’]	
	dlPFC (F2)	H’ 9–10	1	×	0		−		
			2	×	0		−		
			3	×	0		AD	2 s [K’]	
		H’ 8–9	4	×	0		−		
		H’ 7–8	3	×	0		AD	10 s [K’]	
	mPFC	L’3–4	1	×	0		−		
			2	×	0		−		
			3	×	0		−		
			4	+	1		−		✓
			4	+	1		AD	2 s [K’, N′]	
		N’1–2	3	×	0		−		
			4	×	0		−		
		J’1–2	3	×	0		−		
			4	×	0		−		
Cingulate cortex	pACC	G’1–2	3	×	0		−		
			4	×	0		AD	1 s [K′]	
		F’1–2	3	×	0		−		
			4	×	0		−		
	dACC	L’1–2	1	×	0		−		
			2	×	0		−		
			3	+	1		AD	11 s [K′, L’]	
			4	+	1		−		✓
			4	+	1		AD	1 s [K′, L’]	
		H’1–2	3	×	0		AD	5 s [L’]	
			4	×	0		−		
		K’1–2	3	×	0		−		
			4	×	0		−		
	MCC	M’1–2	3	#			−		
Insula	sAI	X’1–2	3	×	0		−		✓
		Y’1–2	3	×	0		−		
	Lim-I	T’1–2	3	×	0		−		

Within each anatomical target, contact pairs are ordered from those eliciting the most prominent electroclinical responses to progressively more distant sites. Within each contact pair, stimulations are listed by ascending order of intensity and, secondarily, chronologically. Clinical responses were classified as follows: +/1: subjective manifestations only; ++/2: subjective and autonomic manifestations with subtle postural adjustments; +++/3: subjective and autonomic manifestations with overt embarrassment-related motor behaviour; #: unusual symptom (Mariani et al., 2021; left-sided head discomfort); ×/0: no clinical manifestations. All stimulations were performed in the supine position unless otherwise indicated (↑), denoting stimulation performed in the upright posture. Trials included in the functional connectivity (FC) analysis are marked with ✓. The trial shown in Figure 2 is indicated with *.

AD, afterdischarges (Mariani et al., 2021); dACC, dorsal anterior cingulate cortex; dlPFC, dorsolateral prefrontal cortex; F2sb, middle frontal gyrus bank of the superior frontal sulcus; F2, middle frontal gyrus; FCD IIa, focal cortical dysplasia type IIa; Lim-I, limen insulae; MCC, middle cingulate cortex; mPFC, mesial prefrontal cortex; pACC, pregenual anterior cingulate cortex; S, seizure; sAI, superior anterior insula.

Stimulation of the F2sb (K′ 8–9, 3 mA; K′ 9–10, 2 and 3 mA) consistently reproduced the electroclinical features of spontaneous seizures (2/2 trials for each condition; Table 1). Although identical stimulation parameters reproduced the subjective and autonomic manifestations under both conditions, the complete behavioural repertoire of embarrassment consistently emerged only during upright stimulation. Specifically, while stimulation in the supine position primarily induced blushing and gaze aversion with subtle postural changes, additional motor features suggestive of embarrassment—such as lowering the head, avoiding eye contact with the observer, and placing the hands in her pockets—were observed only when stimulation was delivered in the upright position (Figure 2). Upon specific questioning, the patient acknowledged that she had behaved as if she were “hiding from people.”

**Figure 2:**
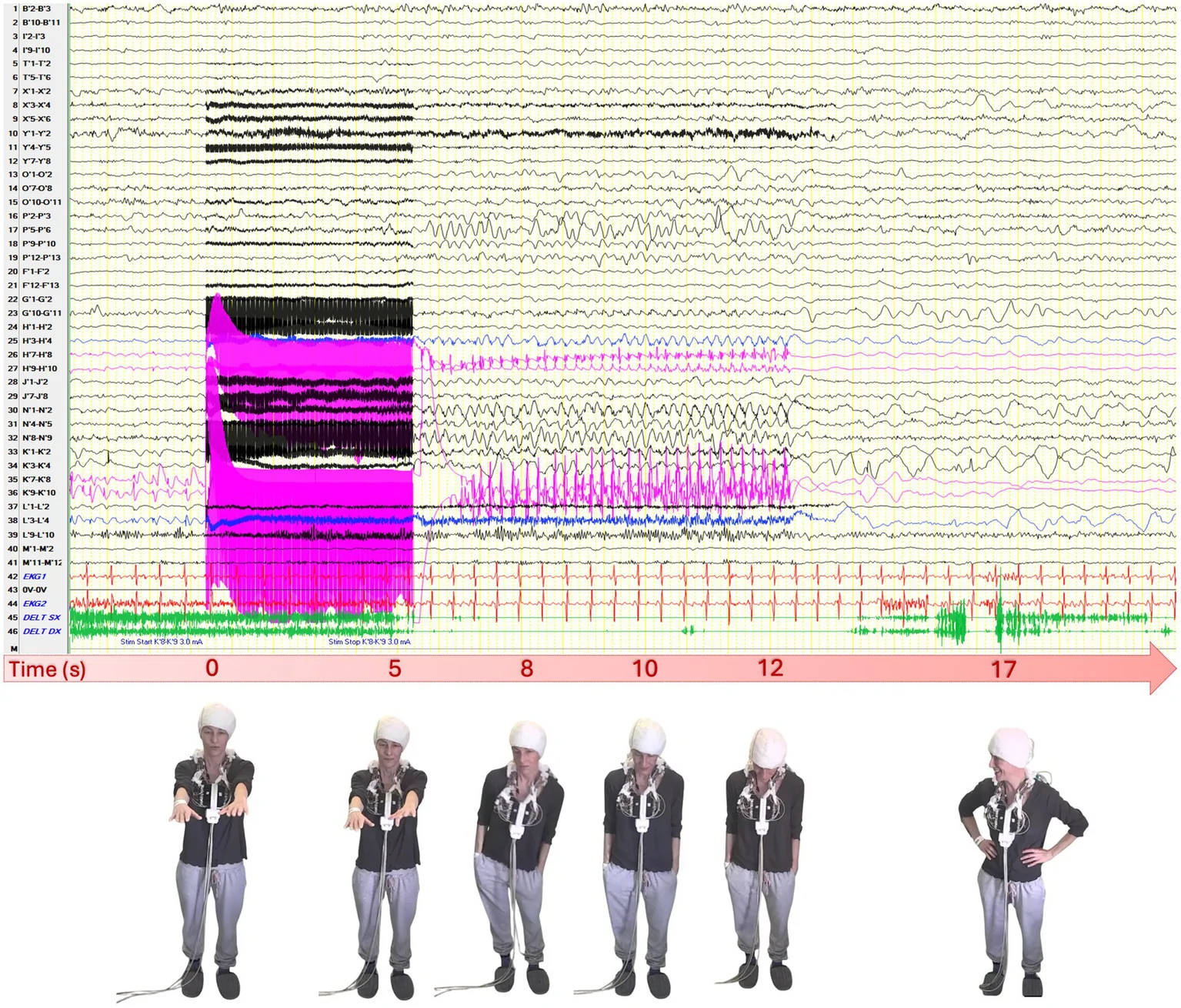
Clinical and electrographic features of stimulation-induced habitual seizure in upright position. Top: stereo-EEG recording of a seizure induced by electrical stimulation of the F2sb (K′ 8–9, 3 mA; filters: 50 𝜇V/mm, TC 0.3, HF 300, 30 s/page). Following the stimulation artifact, rhythmic spike-and-wave (4 Hz) activity appears over the F2sb (K′ 7–10 and adjacent electrode H′ 8–10, shown in pink), accompanied by medium-voltage tonic activity involving predominantly the ACC (L’1–2, shown in blue), mesial prefrontal cortex (L’3–4, shown in blue), and superior AI (Y’1–2). Bottom: sequential clinical changes observed in the upright position, illustrating the typical seizure semiology (timeline in seconds starting from stimulation onset). The episode begins with facial flushing (5 s), followed by the subject placing both hands in her pockets (8 s) and progressively lowering her head (10–12 s). At the end of the event (17 s), she resumes smiling with a confident demeanor, consistent with her baseline attitude.

Electrical stimulation of the dACC (L’ 1–2, 4 mA) and adjacent mesial prefrontal cortex (mPFC; L’ 3–4, 4 mA) elicited mild sensations without objective manifestations.

### 3.3 Functional connectivity analysis

#### 3.3.1 Methods

We analyzed stimulation-induced changes in functional connectivity to assess whether stimulation at different sites produced distinct patterns of network reconfiguration associated with subsequent clinical phenomena.

Regions of interest selected for the analysis were defined according to their involvement during spontaneous seizures and anatomical considerations. For each region, one or, when feasible, two contact pairs were selected based on their maximal ictal recruitment to ensure regional representation across stimulation conditions, as contacts directly involved in stimulation were excluded from the analysis when affected by stimulation artifacts.

Three stimulation conditions were analyzed: stimulation reproducing the habitual electroclinical seizure, stimulation eliciting subjective manifestations only (without afterdischarges; Mariani et al., 2021), and asymptomatic stimulation (without afterdischarges; Mariani et al., 2021). For each stimulation, two 60-s periods were chosen for comparison: a background period immediately preceding the stimulation and a post-stimulation period immediately following the stimulation artifact and encompassing the reported semiology.

Connectivity was estimated using non-linear correlation (h^2^), which quantifies the dependence of signal Y on signal X and computes the temporal lag to estimate delays between signals (Bartolomei et al., 2019). Node strength (i.e., the importance of a given node in the network), defined as the sum of all h^2^ values associated with a given node independently of the direction of the link, was used to summarize connectivity patterns (Bartolomei et al., 2019). We computed all the pairwise h^2^ values using a 3-s sliding window with a 0.5-s step and a maximum delay between signals of 100 ms. Analyses were performed in two frequency bands: broadband (4–45 Hz) and beta (15–30 Hz). Signals were analyzed using AnyWave software (Colombet et al., 2015).

#### 3.3.2 Statistical analysis

Node strength was normalized by node degree (i.e., divided by n − 1, the maximum possible number of connections for a given node). Differences between post- and pre-stimulation conditions were assessed using the Mann–Whitney-Wilcoxon rank-sum test. Statistical significance was assessed using Bonferroni correction across channels, with a family-wise *α* of 0.05. Effect sizes (*r*) were computed from the rank-sum *z*-statistic as *r* = z/√N where N is the total number of observations included in each comparison. A sensitivity analysis using non-overlapping windows (~19 independent windows per period) was performed to control for temporal autocorrelation between overlapping sliding-window estimates.

#### 3.3.3 Results

The functional connectivity changes are reported in the broadband range (4–45 Hz), while the corresponding beta-band analyses and detailed statistical results are provided in Supplementary Table 1.

The typical electrically induced seizure, elicited by F2sb stimulation, was associated with reduced node strength in the dACC, mPFC, dorsolateral prefrontal cortex (dlPFC), and sAI, whereas increased node strength was observed in the limen insulae (Lim-I; Figure 3, panel A). Mild subjective ictal manifestations were associated with decreased node strength in the dACC and sAI following dACC stimulation (Figure 3, panel C), and in the mPFC and dlPFC following mPFC stimulation (Figure 3, panel D). Asymptomatic sAI stimulation was associated with increased node strength in the dACC and Lim-I, together with decreased node strength in the sAI (Figure 3, panel B).

**Figure 3:**
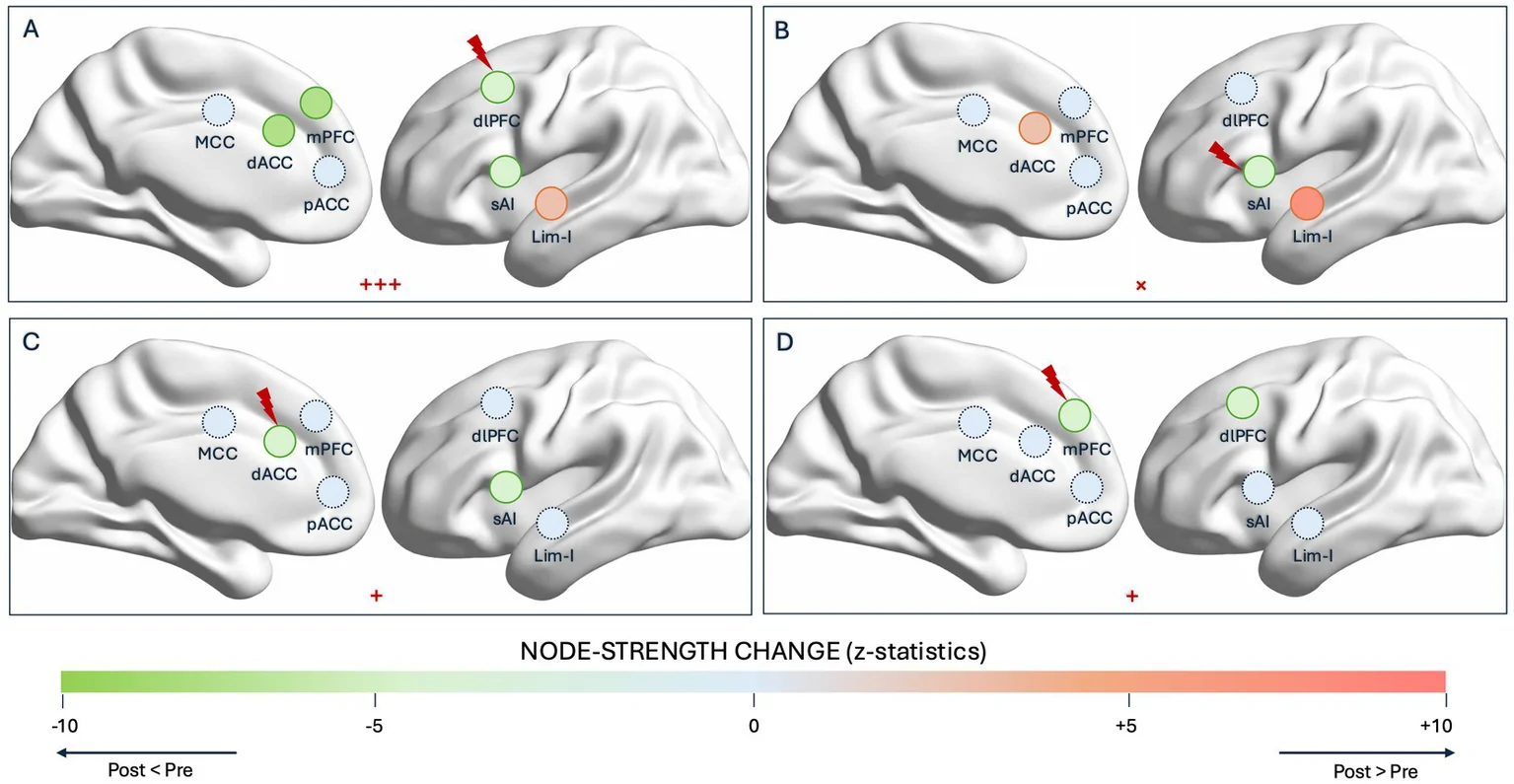
Functional connectivity changes and clinical manifestations across stimulation sites. Non-linear correlation analysis was performed with a montage including contacts in the pregenual ACC (pACC; G’1–2), posterior and dorsal ACC (dACC; L’1–2), middle cingulate cortex (MCC; M’1–2), mesial prefrontal cortex (mPFC; N’1–2), dorsolateral prefrontal cortex (dlPFC; K’9–10 and H’8–9), superior anterior insula (anterior short gyrus; sAI; X’1–2 and Y’1–2), and limen insulae (Lim-I; T’1–2). When more than one contact pair sampled the same anatomical region and neither was involved in stimulation, the pair showing the largest absolute z statistic was represented. Node colors represent the magnitude and direction of post- vs. pre-stimulation node-strength changes (z-scores), with green indicating decreased and red indicating increased node strength. Solid outlines indicate significant differences after Bonferroni correction, whereas light-blue circles with dashed outlines indicate non-significant differences. Red lightning bolts indicate the stimulation site. Clinical responses were classified as follows: +: subjective manifestations only; +++: subjective and autonomic manifestations with overt embarrassment-related motor behaviour; ×: no clinical manifestations. Panels illustrate connectivity changes following: **(A)** dlPFC stimulation (K’9–10, 2 mA), **(B)** sAI stimulation (X’1–2, 3 mA), and **(C,D)** dACC and mPFC stimulation (L’1–2, 4 mA and L’3–4, 4 mA).

##### 3.3.3.1 Sensitivity analysis results

Using a conservative approach based on non-overlapping windows, reduced node strength in the dACC, sAI, and mPFC associated with the typical seizure remained significant. The other broadband effects did not reach significance under this stricter test and should be interpreted as exploratory, given the single-trial nature of these stimulations. Nevertheless, the direction of the effect sizes was largely consistent with that observed in the primary analysis, suggesting that the underlying signal, although not statistically confirmed at this reduced sample size, was not solely artifactual.

### 3.4 Informed consents

The patient provided written informed consent for the use of anonymized clinical and electrophysiological data, including potentially identifiable images for research purposes.

## 4 Clinical outcome

Based on stereo-EEG data, thermocoagulation was performed exclusively in the F2sb, resulting in seizure freedom for 3 months. Subsequently, left frontal cortectomy led to histopathological confirmation of focal cortical dysplasia type IIa (Blümcke et al., 2011) and resulted in seizure freedom at 6-month follow-up.

## 5 Discussion

The present case illustrates a rare ictal semiology characterized by a dissociation between positively valenced emotional experience and the behavioural features typical of embarrassment. Despite reporting intense joy, the patient exhibited blushing, a slumped posture, gaze aversion, and reduced speech, a pattern that she herself later described as an attempt to hide from others (Landers and Sznycer, 2022). The spontaneous seizure originated in the F2sb and rapidly recruited the dACC and sAI, while electrical stimulation of the F2sb reliably reproduced the habitual electroclinical seizure. Strikingly, identical stimulation produced markedly different motor responses depending on body posture, with overt embarrassment emerging only in the upright position. Functional connectivity analyses further indicated that the full-blown behavioural manifestations were associated with reduced node strength within a core network comprising the dACC, sAI, mPFC, and dlPFC. In contrast, more limited network desynchronization following dACC and mPFC stimulation—restricted to the dACC and sAI, or to the mPFC and dlPFC, respectively—was accompanied only by mild subjective symptoms, whereas asymptomatic sAI stimulation elicited a distinct connectivity pattern involving the dACC, Lim-I, and sAI, with the dACC changing in the opposite direction. These findings underscore that ictal phenomenology arises from the transient reconfiguration of distributed neural networks rather than from dysfunction of a single brain region (Bartolomei et al., 2019; Picard and Craig, 2009). While current literature does not provide an established pathophysiological explanation for this phenomenon in people with epilepsy, we discuss how the dACC, sAI, and PFC may collectively contribute to this multifaceted semiology, drawing on their role within the “social brain” as conceptualized in social neuroscience (Della Vedova and Proverbio, 2024).

The ACC and AI—two regions enriched with von Economo neurons (Picard and Craig, 2009)—form an integrated network (Picard and Kurth, 2014) involved in emotions with interpersonal significance, including maternal attachment and empathy (Craig et al. 2009; Caruana et al., 2018; Hedaya and Ver Hoef, 2024; Lavin et al., 2013; Medford and Critchley, 2010; Lieberman, 2007). Within this framework, the AI is thought to generate a unified representation of bodily states by integrating homeostatic signals from the posterior insula with interoceptive and emotional information (Taylor et al., 2009). Activation of the AI could enhance self-awareness and well-being during the widely recognized ecstatic seizures (Bartolomei et al., 2019; Picard and Craig, 2009; Picard and Kurth, 2014). Conversely, our patient denied any egocentric or mystical quality to her emotional experience and simultaneously exhibited behavioural features incongruent with this state, suggesting that insular activation alone cannot fully account for the ictal semiology. Recruitment of the ACC is crucial for coordinating adaptive behavioural strategies in response to feeling states generated by the AI (Perini et al., 2018; Medford and Critchley, 2010). While the ventral ACC is primarily associated with emotion and visceromotor control, the dorsal bank and adjacent cingulate sulcus are implicated in domain-general cognitive processes, including evaluation of motivational content and selection of motor responses during goal-directed behaviour (Devinsky et al., 1995; Caruana et al., 2018). Located at the intersection of affect, cognition, and behaviour, the ACC links emotionally salient stimuli to autonomic responses and regulates context-appropriate motor behaviour (Devinsky et al., 1995; Medford and Critchley, 2010).

Embarrassment typically arises when deviations from personal standards occur under public scrutiny, reflecting heightened attention to personal appearance and social evaluation by others (Crozier and De Jong, 2012). The insula continuously generates interoceptive predictions based on acquired models of social behaviour, updating them in response to incoming sensory information and producing prediction errors when expectations about one’s performance are violated (Bartolomei et al., 2019; Craig et al. 2009; Müller-Pinzler et al., 2015). More recent fMRI evidence has shown the involvement of both the sAI and dACC in processing the salience of being judged by others, irrespective of the quality of this judgment (Perini et al., 2018). Taken together, the sAI and dACC may be conceptualized as core nodes in monitoring self-relevance during social interactions (Perini et al., 2018), constituting key neurobiological substrates for the experience and expression of embarrassment. The additional involvement of the mesial and dorsolateral prefrontal cortices—also reported in another case of ictal embarrassment (Sun et al., 2021)—likely reflects the contribution of higher-order regulatory systems supporting selective attention and response planning (Phillips et al., 2008; Heilbronner and Hayden, 2016). According to this interpretation, transient disruption of top-down prefrontal modulation may permit aberrant activation of the AI-ACC network.

Altered regulatory control during seizures, potentially distorting emotional states, may explain the coexistence of positive emotional experience and embarrassment-related behaviour (Crozier, 1998; Crozier and De Jong, 2012). Although classically associated with negative events, embarrassment may also arise from positive social exposure (Crozier, 1998; Crozier and De Jong, 2012). A complementary explanation for this unusual combination lies in the conflict dimension intrinsic to emotions carrying a moral significance. Moral emotions act as motivational forces providing evaluative feedback and driving behaviour in response to transgressions and prosocial outcomes, with their phenomenology critically shaped by social context (Fourie et al., 2014). Interestingly, their neural correlates include the ACC and AI together with mentalizing regions such as the prefrontal cortex, mirroring the network implicated in our patient (Fourie et al., 2014). Demanding moral dilemmas particularly recruit the mPFC, a key region for self-referential processing that cooperates with the dACC in monitoring competing tendencies between emotional and cognitive processes (Bryant et al., 2016). Even in the absence of an identifiable embarrassing real-world event, as in this case, an internal conflict—potentially reflecting a discrepancy between the internal emotional state and external evaluative cues—may engage the dACC, which could mediate the autonomic response and behavioural adjustment (Bryant et al., 2016).

### 5.1 Limitations

Several limitations should be acknowledged in this work: the single-case design, the exploratory nature of the connectivity analysis, and the absence of quantitative behavioural measures. The potential confounding effect of social exposure could not be independently assessed because, for safety reasons, upright stimulations were not performed with the patient alone in the room.

### 5.2 Conclusion

The unique semiology of this case is characterized by the coexistence of subjective joy and objective features of embarrassment. Stereo-EEG recordings, complemented by exploratory connectivity analyses, revealed transient dysfunction within a distributed network centered on the superior anterior insula, dorsal anterior cingulate cortex, and prefrontal cortex. The dynamic interplay among these regions provides a plausible framework linking the multifaceted dimensions of socially meaningful emotions, including perception, motor-autonomic manifestations, self-monitoring, regulatory control, and moral appraisal. Body posture served as a naturalistic probe, revealing components of embarrassment that remained unapparent during supine stimulation and suggesting that the expression of complex behaviours depends on the opportunities afforded by bodily context.

## 6 Patient perspective

Going through this long evaluation and repeatedly experiencing some of the symptoms I have during my seizures helped me to better understand what happens to me during these episodes. Some of these sensations felt strange and difficult to put into words, but I felt reassured that the medical team listened carefully and tried to make sense of them with me. I hope that sharing my story can help people with epilepsy find meaning in their seizures, as well as their cause and an effective treatment.

## Data Availability

The raw data supporting the conclusions of this article will be made available by the authors, without undue reservation.
