# Supplementary material for: Behavioural expression of ictal embarrassment: a stereo-EEG case report
Source: Front Hum Neurosci. 2026 Oct 6;20:1951958. doi: 10.3389/fnhum.2026.1951958 (PMC13680394; doi:10.3389/fnhum.2026.1951958)
Supplement: Supplementary file 1 [file Data_Sheet_1.pdf]

Condition	Region	Band	z	N (total)	r = z/sqrt(N)	|r|	P-value (nominal)	Direction	Significance (Bonferroni)
A - Typical seizure	Pregenual ACC (G'1-2)	Broadband (4-45 Hz)	0,56	228	0,0371	0,0371	0,576	increase	ns
A - Typical seizure	Pregenual ACC (G'1-2)	Beta (15-30 Hz)	1,14	228	0,0755	0,0755	0,25615	increase	ns
A - Typical seizure	Dorsal ACC (L'1-2)	Broadband (4-45 Hz)	-9,02	228	-0,5974	0,5974	0	decrease	**
A - Typical seizure	Dorsal ACC (L'1-2)	Beta (15-30 Hz)	-7,67	228	-0,508	0,508	0	decrease	**
A - Typical seizure	Superior AI (X'1-2)	Broadband (4-45 Hz)	-0,77	228	-0,051	0,051	0,44364	decrease	ns
A - Typical seizure	Superior AI (X'1-2)	Beta (15-30 Hz)	-5,88	228	-0,3894	0,3894	0	decrease	**
A - Typical seizure	Superior AI (Y'1-2)	Broadband (4-45 Hz)	-3,72	228	-0,2464	0,2464	0,0002	decrease	*
A - Typical seizure	Superior AI (Y'1-2)	Beta (15-30 Hz)	-7,4	228	-0,4901	0,4901	0	decrease	**
A - Typical seizure	Mesial PFC (N'1-2)	Broadband (4-45 Hz)	-6,87	228	-0,455	0,455	0	decrease	**
A - Typical seizure	Mesial PFC (N'1-2)	Beta (15-30 Hz)	-5,96	228	-0,3947	0,3947	0	decrease	**
A - Typical seizure	Dorsal PFC (H'8-9)	Broadband (4-45 Hz)	-3,12	228	-0,2066	0,2066	0,00184	decrease	*
A - Typical seizure	Dorsal PFC (H'8-9)	Beta (15-30 Hz)	-5,81	228	-0,3848	0,3848	0	decrease	**
A - Typical seizure	MCC (M'1-2)	Broadband (4-45 Hz)	-1,02	228	-0,0676	0,0676	0,30912	decrease	ns
A - Typical seizure	MCC (M'1-2)	Beta (15-30 Hz)	-4,3	228	-0,2848	0,2848	0,00002	decrease	**
A - Typical seizure	Limen insulae (T'1-2)	Broadband (4-45 Hz)	3,08	228	0,204	0,204	0,00209	increase	*
A - Typical seizure	Limen insulae (T'1-2)	Beta (15-30 Hz)	-2,84	228	-0,1881	0,1881	0,00453	decrease	ns
B - No sensation	Pregenual ACC (G'1-2)	Broadband (4-45 Hz)	0,53	228	0,0351	0,0351	0,59533	increase	ns
B - No sensation	Pregenual ACC (G'1-2)	Beta (15-30 Hz)	-2,23	228	-0,1477	0,1477	0,02589	decrease	ns
B - No sensation	Dorsal ACC (L'1-2)	Broadband (4-45 Hz)	4,61	228	0,3053	0,3053	0	increase	**
B - No sensation	Dorsal ACC (L'1-2)	Beta (15-30 Hz)	-2,02	228	-0,1338	0,1338	0,04369	decrease	ns
B - No sensation	Superior AI (X'1-2)	Broadband (4-45 Hz)	-3,88	228	-0,257	0,257	0,00011	decrease	*
B - No sensation	Superior AI (X'1-2)	Beta (15-30 Hz)	-11,61	228	-0,7689	0,7689	0	decrease	**
B - No sensation	Superior AI (Y'1-2)	Broadband (4-45 Hz)	-1,89	228	-0,1252	0,1252	0,05895	decrease	ns
B - No sensation	Superior AI (Y'1-2)	Beta (15-30 Hz)	-11,61	228	-0,7689	0,7689	0	decrease	**
B - No sensation	Mesial PFC (N'1-2)	Broadband (4-45 Hz)	0,87	228	0,0576	0,0576	0,38514	increase	ns
B - No sensation	Mesial PFC (N'1-2)	Beta (15-30 Hz)	-2,99	228	-0,198	0,198	0,00282	decrease	ns
B - No sensation	Dorsal PFC (H'8-9)	Broadband (4-45 Hz)	2,47	228	0,1636	0,1636	0,01348	increase	ns
B - No sensation	Dorsal PFC (H'8-9)	Beta (15-30 Hz)	-1,79	228	-0,1185	0,1185	0,07343	decrease	ns
B - No sensation	Dorsal PFC (K'9-10)	Beta (15-30 Hz)	-4,41	228	-0,2921	0,2921	0,00001	decrease	**
B - No sensation	MCC (M'1-2)	Broadband (4-45 Hz)	0,18	228	0,0119	0,0119	0,86053	increase	ns

B - No sensation	MCC (M'1-2)	Beta (15-30 Hz)	-4,51	228	-0,2987	0,2987	0,00001	decrease	**
B - No sensation	Limen insulae (T'1-2)	Broadband (4-45 Hz)	5,55	228	0,3676	0,3676	0	increase	**
B - No sensation	Limen insulae (T'1-2)	Beta (15-30 Hz)	-2,61	228	-0,1729	0,1729	0,00907	decrease	ns
C - Mild, dACC stim	Pregenual ACC (G'1-2)	Broadband (4-45 Hz)	-1,65	228	-0,1093	0,1093	0,09985	decrease	ns
C - Mild, dACC stim	Pregenual ACC (G'1-2)	Beta (15-30 Hz)	-0,98	228	-0,0649	0,0649	0,32663	decrease	ns
C - Mild, dACC stim	Dorsal ACC (L'1-2)	Broadband (4-45 Hz)	-3,68	228	-0,2437	0,2437	0,00023	decrease	*
C - Mild, dACC stim	Dorsal ACC (L'1-2)	Beta (15-30 Hz)	-5,07	228	-0,3358	0,3358	0	decrease	**
C - Mild, dACC stim	Superior AI (X'1-2)	Broadband (4-45 Hz)	-0,57	228	-0,0377	0,0377	0,56645	decrease	ns
C - Mild, dACC stim	Superior AI (X'1-2)	Beta (15-30 Hz)	-0,75	228	-0,0497	0,0497	0,45084	decrease	ns
C - Mild, dACC stim	Superior AI (Y'1-2)	Broadband (4-45 Hz)	-3,1	228	-0,2053	0,2053	0,00191	decrease	*
C - Mild, dACC stim	Superior AI (Y'1-2)	Beta (15-30 Hz)	-0,95	228	-0,0629	0,0629	0,34171	decrease	ns
C - Mild, dACC stim	Mesial PFC (N'1-2)	Broadband (4-45 Hz)	-2,33	228	-0,1543	0,1543	0,0199	decrease	ns
C - Mild, dACC stim	Mesial PFC (N'1-2)	Beta (15-30 Hz)	3,03	228	0,2007	0,2007	0,00245	increase	*
C - Mild, dACC stim	Dorsal PFC (H'8-9)	Broadband (4-45 Hz)	-0,25	228	-0,0166	0,0166	0,79948	decrease	ns
C - Mild, dACC stim	Dorsal PFC (H'8-9)	Beta (15-30 Hz)	0,04	228	0,0026	0,0026	0,96556	increase	ns
C - Mild, dACC stim	Dorsal PFC (K'9-10)	Broadband (4-45 Hz)	1,56	228	0,1033	0,1033	0,11894	increase	ns
C - Mild, dACC stim	Dorsal PFC (K'9-10)	Beta (15-30 Hz)	1,68	228	0,1113	0,1113	0,09341	increase	ns
C - Mild, dACC stim	MCC (M'1-2)	Broadband (4-45 Hz)	2,23	228	0,1477	0,1477	0,02562	increase	ns
C - Mild, dACC stim	MCC (M'1-2)	Beta (15-30 Hz)	2,27	228	0,1503	0,1503	0,02333	increase	ns
C - Mild, dACC stim	Limen insulae (T'1-2)	Broadband (4-45 Hz)	2,43	228	0,1609	0,1609	0,01499	increase	ns
C - Mild, dACC stim	Limen insulae (T'1-2)	Beta (15-30 Hz)	-1,5	228	-0,0993	0,0993	0,1344	decrease	ns
D - Mild, mPFC stim	Pregenual ACC (G'1-2)	Broadband (4-45 Hz)	-0,04	228	-0,0026	0,0026	0,96716	decrease	ns
D - Mild, mPFC stim	Pregenual ACC (G'1-2)	Beta (15-30 Hz)	-1,94	228	-0,1285	0,1285	0,05253	decrease	ns
D - Mild, mPFC stim	Dorsal ACC (L'1-2)	Broadband (4-45 Hz)	-1,32	228	-0,0874	0,0874	0,18674	decrease	ns
D - Mild, mPFC stim	Dorsal ACC (L'1-2)	Beta (15-30 Hz)	0,3	228	0,0199	0,0199	0,76403	increase	ns
D - Mild, mPFC stim	Superior AI (X'1-2)	Broadband (4-45 Hz)	1,07	228	0,0709	0,0709	0,28495	increase	ns
D - Mild, mPFC stim	Superior AI (X'1-2)	Beta (15-30 Hz)	-0,33	228	-0,0219	0,0219	0,74116	decrease	ns
D - Mild, mPFC stim	Superior AI (Y'1-2)	Broadband (4-45 Hz)	0,78	228	0,0517	0,0517	0,43651	increase	ns
D - Mild, mPFC stim	Superior AI (Y'1-2)	Beta (15-30 Hz)	0,36	228	0,0238	0,0238	0,72002	increase	ns
D - Mild, mPFC stim	Mesial PFC (N'1-2)	Broadband (4-45 Hz)	-4,44	228	-0,294	0,294	0,00001	decrease	**
D - Mild, mPFC stim	Mesial PFC (N'1-2)	Beta (15-30 Hz)	-0,34	228	-0,0225	0,0225	0,7351	decrease	ns
D - Mild, mPFC stim	Dorsal PFC (H'8-9)	Broadband (4-45 Hz)	-3,3	228	-0,2185	0,2185	0,00098	decrease	*

D - Mild, mPFC stim	Dorsal PFC (H'8-9)	Beta (15-30 Hz)	-0,7	228	-0,0464	0,0464	0,48657	decrease	ns
D - Mild, mPFC stim	Dorsal PFC (K'9-10)	Broadband (4-45 Hz)	-3,29	228	-0,2179	0,2179	0,00099	decrease	*
D - Mild, mPFC stim	Dorsal PFC (K'9-10)	Beta (15-30 Hz)	-4,04	228	-0,2676	0,2676	0,00005	decrease	**
D - Mild, mPFC stim	MCC (M'1-2)	Broadband (4-45 Hz)	-0,88	228	-0,0583	0,0583	0,37641	decrease	ns
D - Mild, mPFC stim	MCC (M'1-2)	Beta (15-30 Hz)	1,43	228	0,0947	0,0947	0,15309	increase	ns
D - Mild, mPFC stim	Limen insulae (T'1-2)	Broadband (4-45 Hz)	-1,2	228	-0,0795	0,0795	0,22944	decrease	ns
D - Mild, mPFC stim	Limen insulae (T'1-2)	Beta (15-30 Hz)	-0,54	228	-0,0358	0,0358	0,59117	decrease	ns

Supplementary Table 1. Functional connectivity changes following electrical stimulation.

Node-strength changes between the 60-s pre- and post-stimulation periods were assessed using the Mann–Whitney–Wilcoxon rank-sum test. Effect sizes (r) were computed from the rank-sum z-statistic as $r = z/\sqrt{N}$, where N is the total number of pre- and post-stimulation observations included in each comparison (N = 228 for the primary overlapping-window analysis). P values are nominal (uncorrected), whereas statistical significance was assessed using Bonferroni correction across channels, with a family-wise α of 0.05. Significance markers therefore indicate results that remained significant after Bonferroni correction (*corrected $p < 0.05$; **corrected $p < 0.001$). The primary analysis was based on 3-s sliding windows with a 0.5-s step. The sensitivity analysis was performed using non-overlapping windows (~19 windows per 60-s period; approximately N = 38 observations per comparison) to account for temporal dependence between overlapping estimates. Broadband (4–45 Hz) and beta-band (15–30 Hz) results are reported separately. Arrows indicate the direction of the post- versus pre-stimulation node-strength change (↑ increase; ↓ decrease).

Sensitivity analysis, significant results only

	Pregenual ACC (G'1-2)	Dorsal ACC (L'1-2)	Superior AI (X'1-2)	Superior AI (Y'1-2)	Mesial PFC (N'1-2)	Dorsal PFC (H'8-9)	Dorsal PFC (K'9-10)	MCC (M'1-2)	Limen insulae (T'1-2)
A — Typical seizure (K'9-10)	–	▼ broad r=0.62 ** ▼ beta r=0.42 *	▼ beta r=0.35 *	▼ broad r=0.40 * ▼ beta r=0.49 *	▼ broad r=0.43 * ▼ beta r=0.41 *	–	–	▼ beta r=0.37 *	–
B — No sensation (X'1-2)	▼ beta r=0.34 *	–	▼ beta r=0.80 **	▼ beta r=0.78 **	–	–	–	▼ beta r=0.40 *	–
C — Mild - dACC (L'1-2)	–	–	–	–	▲ beta r=0.34 *	–	–	▲ broad r=0.36 *	–
D — Mild - mPFC (L'3-4)	–	–	–	–	–	–	–	–	–
